# Modulating nociception networks: the impact of low-intensity focused ultrasound on thalamocortical connectivity

**DOI:** 10.1093/braincomms/fcaf062

**Published:** 2025-02-08

**Authors:** Arabinda Mishra, Pai-Feng Yang, Thomas J Manuel, Allen T Newton, M Anthony Phipps, Huiwen Luo, Michelle K Sigona, Allison Q Dockum, Jamie L Reed, John C Gore, William A Grissom, Charles F Caskey, Li Min Chen

**Affiliations:** Vanderbilt University Institute of Imaging Science, Vanderbilt University, Nashville, TN, USA; Department of Radiology and Radiological Sciences, Vanderbilt University Medical Center, Nashville, TN, USA; Vanderbilt University Institute of Imaging Science, Vanderbilt University, Nashville, TN, USA; Department of Radiology and Radiological Sciences, Vanderbilt University Medical Center, Nashville, TN, USA; Department of Biomedical Engineering, Vanderbilt University, Nashville, TN, USA; Vanderbilt University Institute of Imaging Science, Vanderbilt University, Nashville, TN, USA; Department of Radiology and Radiological Sciences, Vanderbilt University Medical Center, Nashville, TN, USA; Vanderbilt University Institute of Imaging Science, Vanderbilt University, Nashville, TN, USA; Department of Radiology and Radiological Sciences, Vanderbilt University Medical Center, Nashville, TN, USA; Vanderbilt University Institute of Imaging Science, Vanderbilt University, Nashville, TN, USA; Department of Biomedical Engineering, Vanderbilt University, Nashville, TN, USA; Vanderbilt University Institute of Imaging Science, Vanderbilt University, Nashville, TN, USA; Department of Biomedical Engineering, Vanderbilt University, Nashville, TN, USA; Vanderbilt University Institute of Imaging Science, Vanderbilt University, Nashville, TN, USA; Department of Biomedical Engineering, Vanderbilt University, Nashville, TN, USA; Vanderbilt University Institute of Imaging Science, Vanderbilt University, Nashville, TN, USA; Department of Radiology and Radiological Sciences, Vanderbilt University Medical Center, Nashville, TN, USA; Vanderbilt University Institute of Imaging Science, Vanderbilt University, Nashville, TN, USA; Department of Radiology and Radiological Sciences, Vanderbilt University Medical Center, Nashville, TN, USA; Department of Biomedical Engineering, Vanderbilt University, Nashville, TN, USA; Vanderbilt University Institute of Imaging Science, Vanderbilt University, Nashville, TN, USA; Department of Biomedical Engineering, Vanderbilt University, Nashville, TN, USA; Vanderbilt University Institute of Imaging Science, Vanderbilt University, Nashville, TN, USA; Department of Radiology and Radiological Sciences, Vanderbilt University Medical Center, Nashville, TN, USA; Department of Biomedical Engineering, Vanderbilt University, Nashville, TN, USA; Vanderbilt University Institute of Imaging Science, Vanderbilt University, Nashville, TN, USA; Department of Radiology and Radiological Sciences, Vanderbilt University Medical Center, Nashville, TN, USA; Department of Biomedical Engineering, Vanderbilt University, Nashville, TN, USA

**Keywords:** transcranial focused ultrasound, fMRI, neuromodulation, pain network, somatosensory

## Abstract

Pain engages multiple brain networks, with the thalamus serving as a critical subcortical hub. This study aims to explore the effects of low-intensity transcranial focused ultrasound-induced suppression on the organization of thalamocortical nociceptive networks. We employed MR-guided focused ultrasound, a potential non-invasive therapy, with real-time ultrasound beam localization feedback and fMRI monitoring. We first functionally identified the focused ultrasound target at the thalamic ventroposterior lateral nucleus by mapping the whole-brain blood oxygenation level-dependent responses to nociceptive heat stimulation of the hand using fMRI in each individual macaque monkey under light anaesthesia. The blood oxygenation level-dependent fMRI signals from the heat-responsive thalamic ventroposterior lateral nucleus were analysed to derive thalamocortical effective functional connectivity network using the psychophysical interaction method. Nineteen cortical regions across sensorimotor, cognitive, associative and limbic networks exhibited strong effective functional connectivity to the thalamic ventroposterior lateral during heat nociceptive processing. Focused ultrasound-induced suppression of heat activity in the thalamic ventroposterior lateral nucleus altered nociceptive responses in most of the 19 regions. Data-driven hierarchical clustering analyses of blood oxygenation level-dependent time courses across all thalamocortical region-of-interest pairs identified two effective functional connectivity subnetworks. The concurrent suppression of thalamic heat response with focused ultrasound reorganized these subnetworks and modified thalamocortical connection strength. Our findings suggest that the thalamic ventroposterior lateral nucleus has extensive and causal connections to a wide array of cortical areas during nociceptive processing. The combination of MR-guided focused ultrasound with fMRI enables precise dissection and modulation of nociceptive networks in the brain, a capability that no other device-based neuromodulation methods have achieved. This presents a promising non-invasive tool for modulating pain networks with profound clinical relevance. The robust modulation of nociceptive effective functional connectivity networks by focused ultrasound strongly supports the thalamic ventroposterior lateral as a viable target for pain management strategies.

## Teaser

Transcranial focused ultrasound has emerged as a promising tool for non-invasive pain relief. Mishra *et al*. report that focused ultrasound-induced suppression of thalamic ventroposterolateral nucleus activity reduced nociceptive responses across various cortical regions, including those linked to sensorimotor function and cognition, and reorganized the ventroposterolateral nociceptive effective connectivity networks.

## Graphical Abstract

**graphical abstract:**
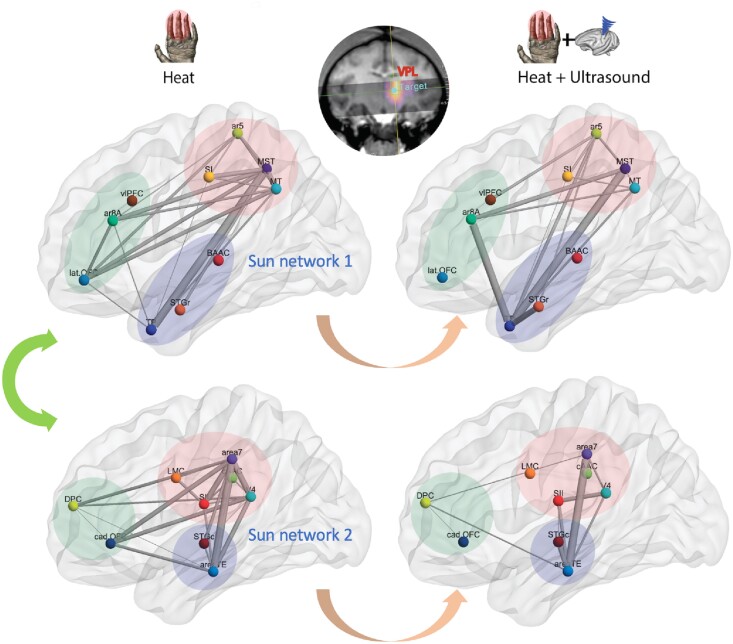

## Introduction

Both acute pain and chronic pain are mediated by coordinated activity across a wide range of brain regions and networks. fMRI studies on human pain networks have delineated a complex pain matrix comprising brain regions and networks that extend beyond those involved in processing and encoding sensory pain experiences.^1-3^ Prior studies by our group and others have shown that many of these putative pain regions, identified in conscious human subjects and patients, also exhibit significant fMRI signal changes under anaesthesia in animal models during the processing of various nociceptive inputs.^4-6^ These regions, often referred to as the nociception matrix, maintain their involvement even under anaesthesia, underscoring their fundamental roles in processing and integrating nociceptive information. This makes them suitable models for further exploration of their causal interconnections using neuromodulation tools that are not readily available in humans.^7-12^

Among the brain regions engaged in nociception, the thalamus serves as a key hub linking subcortical regions to the neocortex. The thalamus plays a critical role in pain perception, in both health and chronic pain conditions. For instance, anatomical tracing and electrophysiology studies in non-human primates (NHPs) have identified nociceptive activity or ascending spinothalamic tracts (SSTs) afferents in several thalamic nuclei, including ventroposterior lateral (VPL) nucleus, ventral posterior inferior nucleus, central lateral nucleus, medial part of the posterior complex, posterior part of the ventral medial nucleus, mediodorsal (MD) nucleus, parafascicular (Pf) nuclei, midline thalamic nuclei (such as submedius nucleus) and intralaminar thalamic nuclei.^13^

Resting-state functional connectivity analyses have revealed the thalamus’s role as a core hub connecting to pain subnetworks.^14^ Our own submillimetre resolution fMRI studies have identified several thalamic nuclei that are responsive to nociceptive heat stimulation of hands in anaesthetized monkeys,^15^ although their links to pain perception remain to be determined. Various thalamic nuclei are involved in pain processing and have been targeted for device-based pain relief methods, such as deep brain stimulation (DBS). However, the clinical benefits of these invasive approaches remain elusive, partly due to the lack of tools that allow high-precision and comparative network perturbation, as well as real-time monitoring of neuromodulation effects. The roles of various thalamic nuclei and their effective functional connections to the cortex remain largely unexplored. Therefore, enhancing our understanding of the organization of thalamocortical nociceptive networks is essential for designing effective therapeutic interventions.

In recent years, low-intensity transcranial focused ultrasound (FUS), which operates through mechanisms entirely different from those of ablative high-intensity FUS, has emerged as a non-invasive neuromodulation tool with unique capabilities. When paired with MRI, low-intensity FUS can access deep brain structures, enabling high-precision targeting and modulation (within a few millimetres) with real-time functional feedback. A recent proof-of-concept study by Badran *et al*.^16^ demonstrated that FUS stimulation of the anterior thalamus altered the pain detection threshold in healthy volunteers. However, the circuit mechanisms underlying FUS action remain uncertain, as actual target engagement and brain network responses were not monitored and evaluated. By combining FUS with MRI, we recently demonstrated in the NHP brain that concurrent FUS suppression of nociceptive heat-evoked blood oxygenation level-dependent (BOLD) fMRI signal in the thalamic VPL nucleus significantly disrupted information processing flow within the nociceptive network.^17^ Together, these clinical and preclinical studies indicate the potential of low-intensity FUS as a clinically relevant tool for pain management.

The current study aims to harness the capabilities of MR-guided FUS (MRgFUS) to dissect nociceptive networks, probe causal thalamocortical functional connections and explore the impacts of suppression of thalamic VPL nucleus on whole-brain heat nociceptive responses as well as network organization. This work builds upon our recent demonstration that FUS can exert bidirectional (excitation and suppression), state- and dose-dependent neuromodulation.^8,9^ Our objectives are to (i) identify cortical regions that effectively connect to thalamic VPL, (ii) establish the thalamocortical effective functional connectivity (EFC) network during the processing of nociceptive heat inputs and (iii) investigate the effects of low intensity focused ultrasound (LIFU) modulatory of thalamic nociceptive response on EFC network organization. EFC measures, derived from EEG^18,19^ or fMRI signals,^19,20^ have been used to infer directed information flow and functional connectivity under task conditions.^21^ We adopted a psychophysiological interaction (PPI) model^22^ to derive EFC measures and implemented a data-driven, unsupervised hierarchical clustering analysis^23^ to classify thalamocortical nociceptive EFC networks and the effects of FUS suppression.^6,24^ Using this network analysis, we found that low-intensity FUS applied to the thalamus induced robust reorganization of nociceptive thalamocortical networks. We used the macaque monkey brain as our model system in this study due to its close anatomical and functional resemblance to the human brain. Our team has extensively mapped the nociceptive networks in this species.^17^

## Materials and methods

### Animal preparation

Four adult macaque monkeys (two female *Macaca fascicularis* and two male *Macaca mulatta*; average age = 10 ± 3 years; average weight = 7.5 ± 2.5 kg) participated in five MRI and FUS neuromodulation experimental sessions. Three animals underwent one session each, and one animal underwent two sessions. For each experimental session, the animals were initially sedated with ketamine hydrochloride (10 mg/kg) and atropine sulphate (0.05 mg/kg) and then anaesthetized using isoflurane (1.0–1.5%) delivered in oxygen. After intubation, each animal’s head was secured in a custom-designed MR stereotaxic frame with ear bars, eye bars and a mouthpiece. During fMRI data acquisition, anaesthesia was carefully monitored and maintained at levels of 1–1.2% isoflurane. The animals were artificially ventilated for the duration of the experiment. The respiration pattern and vital signals (heart rate, end-tidal CO_2_ and SpO_2_) were monitored via SurgiVet equipment, and adjustments were made as needed for the physiological state to remain stable. All procedures adhered to the National Institutes of Health guidelines and were approved by the Institutional Animal Care and Use Committee of Vanderbilt University.

### FUS parameters and presentation paradigm

**Figure 1:**
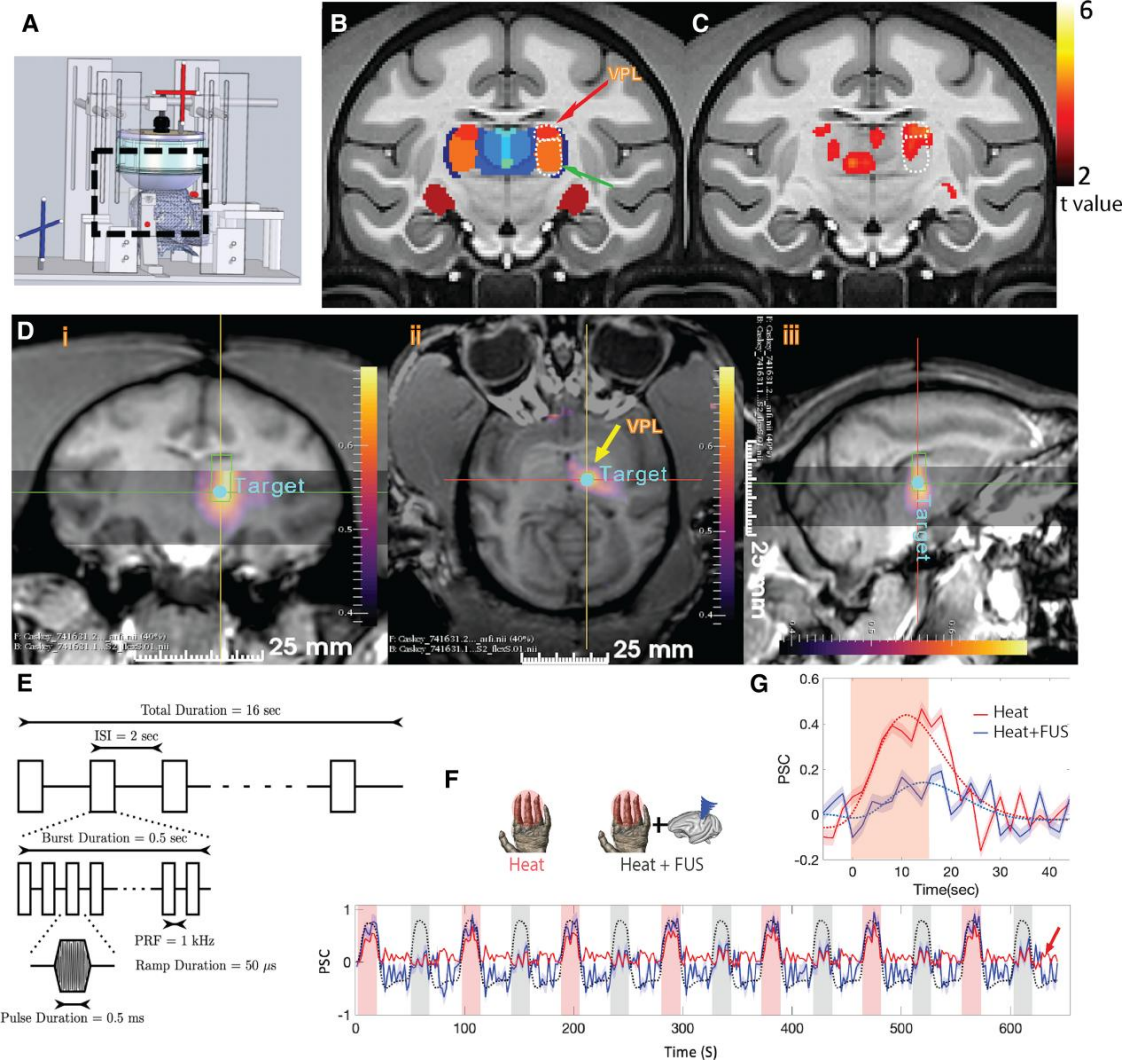
**Targeted FUS stimulation of thalamic VPL and surrounding nuclei with MR-ARFI feedback.** (**A**) Schematic illustration of the experimental set-up, including the fiducials (cross bars) for optical tracking, 128-element transducer and 3D reconstructed monkey head positioned in customized MRI-compatible stereotaxic frame. (**B**) One selected coronal image of the macaque brain atlas (NMT2.0) showing thalamic nuclei, including VPL and ventral lateral posteroventral nucleus (green arrow) highlighted by dotted lines. (**C**) Normalized group nociceptive heat (47.5°C) stimulus-evoked fMRI activation foci are displayed on the corresponding coronal atlas image. Activation foci are thresholded at *t* > 2, *P* < 0.05, FDR corrected (*n* = 24). (**D**) Display of MR-ARFI foci targeted at the thalamic VPL on coronal (i), axial (ii) and sagittal (iii) planes. Scale: tissue displacement in microns. (**E**) A 128-channel FUS array delivered 650 kHz pulsed US with a fast PRF of 1 kHz with a 50% duty cycle for 500 ms and a slow PRF of 0.5 Hz for 16 s. The estimated *in vivo* pressure was 550 kPa (39% transmission estimate). (**F**) PPI regressor (red) where alternate cycles refer to the two regressors (heat and heat + FUS) used in the GLM. (**G**) Cycle-averaged PSC and standard error (shaded band) at the VPL nucleus. The dotted curves present the overall variation of signal change using double gamma fitting.

In the current study, we employed a custom-designed, MR-compatible, 128-element transducer [radius of curvature = 72 mm, outer diameter = 103 mm, element diameter = 6.6 mm, free-field full-width half maximum = 9.3 and 2.2 mm in the axial and lateral directions, respectively,^25^ manufactured by Imasonic (Besancon, France)]. A driving system (Image Guided Therapy, Pessac, France) controlled the sonication parameters. The transducer was positioned around the midline of the head, as illustrated in Fig. 1A. The scalp was shaved, and transmission gel was applied to the scalp to ensure optimal acoustic coupling. A custom water cone was filled with a degassed solution of ∼90% D_2_O and 10% H_2_O. The D_2_O was used to replace most of the H_2_O to reduce signal intensities and geometric distortions in MR images due to ineffective shimming associated with the water concentration in the cone.

The FUS stimulation blocks consisted of trains of 650 kHz pulses,^26^ 500 ms pulse train duration, pulse repetition frequency (PRF) of 1 kHz and pulse duration of 0.5 ms. Detailed FUS parameters are reported in Supplementary Tables 1–4. This pulse train was repeated at 0.5 Hz for 16 s, followed by a 30 s rest period. Individual pulses were ramped up and down (ramp duration 50 μs) to reduce the spectral content in the auditory range.^14,27^ Our target *in vivo* pressure was 550 kPa. For each targeted location, the free-field pressure was estimated using a calibrated hydrophone (HNC 0400, Onda Corp., Sunnyvale, CA, USA). Calibrations of the transducer were performed to compensate for pressure reductions during steering by steering the beam in 1-mm steps of lateral steering out to 7 mm at low pressure (MI < 1), and steering compensation was applied based on the total steered distance. Based on previous water tank measurements with an *ex vivo* NHP skull cap, we estimated transmission to be 39% of the free-field value. Figure 1E provides a schematic overview of the FUS parameters. We have previously demonstrated that the FUS parameters in this study reduce the BOLD signal in the sonicated region during natural tactile stimulation of the circuit.^7-9^

Ultrasound simulations were performed to estimate *in vivo* pressures from the driving free-field pressure. All simulations were performed using the MATLAB acoustic toolbox, k-Wave^28^ and followed methods detailed in previous work.^29^ Ultrasound pulses were simulated as five-cycle sine waves, with the phases corresponding to the therapeutic steering coordinates and amplitudes associated with the free-field pressure. Simulations were performed with a grid size and a voxel size of 0.25 mm. Subject CTs were registered to MR image space using the Slicer software and its ‘General Registration (BRAINS)’ module.^30^ The CT data were used to estimate the speed of sound, density and attenuation through the skull.^31^ Estimates of temperature deposition were simulated in k-Wave using the kWaveDiffusion class. The voxel sizes of thermal simulations were the same as those used for pressure simulations, and heterogeneous medium parameters were used based on CT images as described in Supplementary Materials (Supplementary Tables 1–5).

The transducer was aligned in image space using optical tracking and MR-visible fiducials. Pressure amplitudes were recorded throughout the simulation grid. A dilated skull mask from the subject’s CT was used to determine the maximum pressure amplitude in the brain, rather than the absolute maximum pressure, which is likely to occur in the skull.^25^ The *in vivo* peak negative pressures were estimated to be between 761.75 and 902.74 kPa based on simulations (see Supplementary Materials for full simulation results). We calculated the thermal index for cranial bone (TIC) to be 5.6, as follows: The total highest electrical power of the multi-channel amplifier was 11.1 W, transmitted via a matching network to a transducer with a reported efficiency of 60%. The duty cycle of the pulse during the 16-s neuromodulation block was 12.5%, resulting in a time-averaged power of 0.83 W. The transducer was ∼5.6 cm away from the skull, resulting in an equivalent active aperture of 3.7 cm. Therefore, the TIC is calculated as (1000*11.1*0.6*0.125)/(40*3.7) = 5.6 (shown in Supplementary Fig. 2). For TIC values in the range of 5.0–6.0, ITRUSST recommends limiting sonication duration to 10 s. Because our 16-s sonication exceeds this duration, we performed additional thermal simulations, which revealed an estimated 0.25°C rise in the skull during the 16-s block (Supplementary Fig. 1 and parameters in Supplementary Table 5). We therefore conclude that thermal rise induced by the ultrasound beam does not exceed 2°C and presents non-significant risk.

### MRI data acquisition

Four monkeys underwent five MRI and FUS stimulation sessions. MRI scans were conducted using a Philips 3T Ingenia CX, equipped with a pair of FLEX surface coils (inner diameter = 12 cm) positioned on either side of the head. We acquired three types of MR images:

1. Whole-brain high-resolution T_1_-weighted images (Echo Time (TE) = 4.6 ms, Repetition Time (TR) = 9.9 ms, 500 × 512 × 512 matrix, voxel size = 0.5 × 0.49 × 0.49 mm^3^ and flip angle = 8^°^): These images were primarily used for registering normalized Echo Planar Image (EPI) data in a template space. To correct for phase variations typically caused by motion, an extra navigator echo with no phase encoding was collected before acquiring the image data.
2. MR-acoustic radiation force imaging (ARFI): MR-ARFI measures transient tissue displacements induced by a brief FUS pulse,^25^ offering a real-time visualization of the sonication field. For the MR-ARFI data acquisition, we implemented sonication at a frequency of 650 kHz for 8.5 ms, with a maximum estimated *in vivo* pressure of 3.5 MPa and a duty cycle of 0.85%. Our previous research^25,32^ described in detail the equations that relate these image volumes (optically tracked FUS beam, MR-ARFI displacement image and anatomic brain image). Displacement images were acquired through a 3D spin-echo MRI acquisition (130 × 130 × 16 mm^3^ FOV; acquisition matrix of 65 × 65 × 4; 2.0 × 2.0 × 4.0 mm^3^ acquired voxel size; 112 × 112 × 4 reconstruction matrix; 1.16 × 1.16 × 4 mm^3^ reconstructed voxel size; TE/TR 34/500 ms). To generate ARFI contrast, we used unipolar motion-encoding gradients (with a duration of 8 ms and a strength of 40 mT•m^−1^) aligned parallel to the ultrasound beam. The centre of the image volume was placed near the estimated focus location. Displacement images were reconstructed by subtracting complex phases of four phase images, acquired with switched polarity motion-encoding gradients, both with and without sonication. Reconstruction was performed offline using MATLAB 2020a (MathWorks, Natick, MA, USA). These images were acquired in an interleaved fashion, yielding a total scan duration of 6.0 min for generating a single displacement image. Further details regarding MR-ARFI beam mapping can be found in our earlier publications and others.^33-36^ Figure 1D(i and ii) presents coronal, axial and sagittal views of MR-ARFI targeting the thalamic nuclei (indicated by a yellow arrow), where the colour intensity corresponds to tissue displacement measured in microns.
3. fMRI data: BOLD fMRI time-series data were acquired using a single-shot Gradient echo EPI (GE-EPI) sequence (TE = 30 ms, TR = 2 s, voxel size = 1.43 × 1.43 × 2 mm^3^, matrix size = 112 × 112 × 36). Each fMRI run consisted of 337 imaging volumes. Typically, three to four runs were acquired per session, but two of the animals underwent longer sessions with eight runs. Out of 27 runs (*n* = 4, animals), three were excluded due to severe motion artefacts and a low signal-to-noise ratio, leaving 24 runs for the final fMRI EFC analysis. The median fMRI (EPI) image was registered to higher-resolution T_1_-weighted images acquired in the same geometry. These images were then registered to the template (T_1_-weighted) from a macaque monkey brain MRI atlas (NIMH Macaque Template, NMT v2.0),^37,38^ and the transformations were applied to EPI images.

### Peripheral nociceptive heat stimulation protocol

Fingers were secured using a custom-built hand/finger holder, positioning the palm facing upwards and exposing the glabrous skin of the fingers for stimulation. A CHEPS thermal probe (30 mm in diameter, Medoc) was used to deliver nociceptive heat stimuli at 47.5°C to the fingers. Each fMRI stimulation run comprised three stimulus conditions: FUS only, heat only and heat + FUS (targeting the thalamic nuclei). These conditions were presented in 16-s blocks, interleaved with 30-s rest periods, followed by a randomized sequence. Each stimulation condition was repeated seven times. For this analysis, only blocks consisting of heat and heat + FUS stimulation were considered, as the FUS only condition falls outside the focus of the current study, and we have reported the effects of FUS only from these subjects previously.^9^ During the baseline periods, the thermal probe remained in contact with the skin of the fingers at a temperature of 32°C. A custom microcontroller programme coordinated the pulsing for alternating heat and heat + FUS stimulation.

### Ensuring precise FUS modulation delivery via MRI-ARFI

Achieving precise delivery of FUS modulation to the intended target is critical for our study. To ensure that the acoustic focus is accurately delivered to the designated target—the thalamic nuclei responsive to nociceptive heat stimulation of the hand—we implemented a three-step procedure before applying FUS modulation.

Prior to the FUS neuromodulation study, an fMRI mapping session was conducted to functionally identify the heat-responsive thalamic nuclei within each animal. Figure 1B and C shows representative coronal slices depicting the thalamic nuclei (including the ventroposterior medial and lateral nuclei, indicated by the orange patches) defined on the NMT atlas and BOLD activation detected within the thalamus. During the day of FUS, optical tracking was deployed to guide the FUS beam to the predefined heat-responsive nuclei (Fig. 1A–B). This tracking process occurred outside the MRI scanner using six 15-mm MRI-visible fiducial markers (MM3002, IZI Medical Products, Owings Mills, MD, USA) placed on the stereotactic frame—one on each eye bar and two on each of the two ear bars. A 3D T_1_-weighted image was used to locate the fiducial markers, which helped align imaging plans for MR-ARFI data acquisition. The transducer’s position was tracked with an infrared stereocamera (NDI Polaris, Ontario, Canada) and registered in physical space relative to the fixed positions of fiducials in the imaging space. Later, an MR image-based method was used to place the transducer in a similar position over the midline of the head, with MR-visible fiducials used to infer the focus location.^39^ Finally, we used MR-ARFI mapping to ensure the precise location and accurate delivery of FUS before data acquisition (Fig. 1D(i–iii)). By combining optical tracking and MRI-ARFI, we can confidently steer the FUS beam to the targeted thalamic VPL nucleus, which exhibits heat-evoked BOLD fMRI activation (Fig. 1C). On two occasions, the MR-ARFI displacement was lower than expected, so the FUS pressure was increased to compensate for the higher estimated attenuation. This pressure compensation was also applied to neuromodulation pulses.

### Annotation of thalamic nuclei modulated by FUS using MR-ARFI

**Figure 2:**
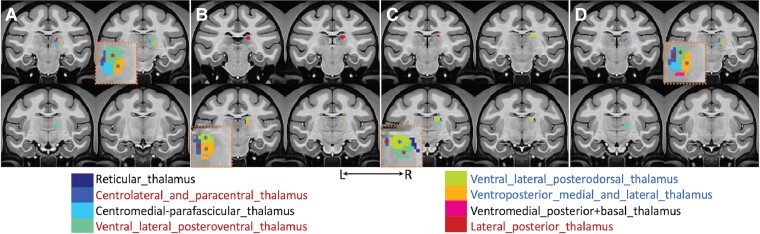
**Annotation of MR-ARFI targets in four representative subjects.** The MR-ARFI focus is overlaid on coronal images of the NMT2.0 template in each subject (**A–D**). The T_1_-weighted anatomical images of four representative subjects have been normalized to fit within the template space. Thalamic nuclei targeted by the MR-ARFI focus, outlined by a circle with a 3-mm diameter, are listed at the bottom of the representative MRI images in the template space. Asterisks (*) shown on the inserted zoom-in images represent thalamic nuclei where statistically significant heat (47.5°C)-evoked fMRI activations were detected. Colours assigned to each thalamic nucleus indicate the frequency with which these nuclei were stimulated by the FUS beam: red (*) refers to all four subjects (4 of 4) and blue (*) refers to three out of four subjects (3 of 4).

We used the NMTv2.0 macaque monkey atlas to annotate the thalamic nuclei targeted by the MR-ARFI beam. According to the atlas, the thalamus is a compact structure composed of 18 major nuclei. To precisely confirm that the VPL nucleus was modulated by FUS, we co-registered the MR-ARFI map acquired in each subject to the brain atlas template for group analysis. The process involved three steps: (i) creating a binary mask of a 3-mm-diameter circle centred around the centre of focus within the MR-ARFI map to define the effective modulation zone in each subject’s native T_1_-weighted image space; (ii) registering the MR-ARFI mask to the brain template by applying transformations (including both rigid and non-rigid transformation matrices) used in registering the anatomical image to the template; and (iii) delineating various thalamic nuclei in the template using the transformed mask. Figure 2 shows the thalamic nuclei within a 3-mm diameter of the MRI-ARFI pressure fields obtained from all four subjects. In all sessions, the FUS sonication beam interacted with multiple nuclei. The inset below the image reports the nuclei most affected across all animals and sessions using frequency maps. The contralateral ventrolateral posteroventral, paracentral and lateral posterior thalamic nuclei were consistently modulated. The analysis has been confined to VPL nucleus.

### FMRI data analysis

#### Pre-processing

The fMRI runs underwent slice timing and 3D motion correction. Six motion parameters were identified, along with time-varying BOLD signals extracted from voxels in white matter and CSF regions that account for at least 70% of the cumulative variance (derived from principal components analysis). These parameters were considered nuisance variables and consequently regressed out. The first five EPI image volumes (equivalent to 10 s of the fMRI time series) were discarded. The median EPI image in each run was co-registered (using a rigid affine transformation) with the T-weighted anatomical image within each subject’s space. This transformation was then applied to the fMRI time series. No spatial smoothing was performed. The BOLD time series were temporally filtered using a Chebyshev Type II filter with a low-pass cut-off frequency of 0.25 Hz. For each subject, the T1-weighted image was co-registered (using a non-rigid transformation) with the macaque template,^37,38^ and this transformation was subsequently applied to the fMRI EPI images. Group-level analysis was performed in template space. The standard pre-processing and normalization of fMRI data to the template were implemented using FSL (*mcflirt, slicetimer, flirt, fnirt*) and custom MATLAB code (R2019b).

#### Detection of stimulus-driven activations

We identified areas of stimulus-driven activation by conducting voxel-wise analyses of BOLD time series using a generalized linear model (GLM). The stimulus paradigm, convolved with the haemodynamic response function (HRF), was used as a predictor to model the stimulus-driven BOLD activation. Voxels demonstrating significant BOLD signal changes (*t* > 2, *P* < 0.05, False Discovery Rate (FDR) corrected) during heat (of hand) and heat + FUS stimulation (of the thalamus) with a minimum of two contiguous EPI voxels were considered as activated and were overlaid on the NMT template for display. Figure 1B shows the colour-coded subregions (nuclei) of the thalamus on a representative coronal slice. The activation *t*-map, overlaid on the anatomic image, illustrates the heat-activated subregions at the group level. Figure 1G presents plots of the cycle-averaged time courses of percentage signal change (PSC) at the thalamic VPL nucleus during heat and heat + FUS stimulation periods. A double gamma fitting (dotted line) shows the overall reduction of PSC when FUS was concurrently delivered with heat stimulation. Automatic identification of thalamic nuclei and cortical areas was performed using custom MATLAB code and AFNI in the template space.

### EFC analysis

EFC, measured using a psychophysiological interaction (PPI) regressor, is predicated on the hypothesis that when two or more functionally active regions interact, their signals exhibit strong correlations during the active period (stimulus on) compared with a passive baseline stimulus-off period.^21,22,40^ The stimulus condition-specific changes in the correlation between the time courses of thalamic nuclei and cortical areas were identified by modelling the stimulus-on and stimulus-off periods separately.^22^ To generate the PPI regressor, the mean PCS was extracted from voxels within the seed region of interest (ROI) (VPL nucleus) that exhibited stimulus-evoked BOLD signal changes, thresholded at *t* > 2 (*P* < 0.05, FDR corrected), in the template space (*n* = 24 runs of four animals). The extracted signal was detrended by removing the linear trend (using the best straight-line fit) and then demeaned subtracting the mean signal (blue line in Fig. 1D). The demeaned task time course was convolved with a double gamma fitted HRF to produce the task regressor (dotted black line). This convolved signal was multiplied element by element with the seed time course (blue line) to generate the PPI regressor. An example of the resulting PPI regressor (red line) is shown in Fig. 1D, illustrating the product of the task-convolved HRF (dotted black line) and the seed time course (blue line). Therefore, the PPI regressor essentially correlates with the task and anti-correlates with the rest period in the EFC analysis. Finally, two regressors were created by concatenating the respective stimulus cycles (stimulus + rest periods) for each condition (heat and heat + FUS) for the GLM analysis.

### Statistical analysis

Group-level analysis in the template space was performed by generating regression coefficients for both heat and heat + FUS conditions. Voxel-wise analyses of the time courses modulated with the stimulus presentation paradigm within the VPL were used for the GLM. Each run was analysed separately for the two stimulus conditions. For group data analysis, we used a higher-level mixed-effects model, which accounts for both within-subject variability, arising from multiple runs, and between-subject variability. Voxels that showed strongly correlated activity (indicated by greater beta values) delineated using both regressors for heat or heat + FUS conditions were used to generate a group-level *t*-map, which was thresholded at a statistically significant level of *t* > 2 (*P* < 0.05, FDR corrected) with a minimum of two contiguous EPI voxels. Repeated measures from multiple runs are treated as a random effect.

Nineteen cortical ROIs exhibited strong EFC to the thalamic VPL nucleus during heat stimulation, and 15 out of the 19 retained EFC during heat + FUS stimulation conditions. The changes in EFC at each node (ROIs) due to the presence of FUS modulation of the thalamic nucleus were evaluated using regression coefficients (beta values). The statistical significance of the difference between the heat and heat + FUS conditions was measured using the non-parametric Mann–Whitney–Wilcoxon (MWW) test. Visualization and interpretation of EFC on inflated hemispheres were performed using SUMA (https://afni.nimh.nih.gov/Suma).

### Hierarchical clustering analysis

To gain a deeper understanding of the functional organization of the EFC networks under both (heat and heat + FUS) stimulus conditions, the PSC and parameters associated with a modified double gamma function^41^ were input into a hierarchical clustering analysis. The modified gamma model fits the stimulus cycle-averaged PSCs with two-gamma functions and derives parameters such as amplitude coefficient and time lags. In addition, we also added the mean beta value (regression coefficients) as an input vector, which directly indicates functional connectivity of each ROI with VPL nuclei. The hierarchical clustering partitions the input data into a multi-level cluster tree (dendrogram) that classifies thalamocortical ROI pairs into subnetworks corresponding to various levels of hierarchy within the EFC network.^23,42^ The classification was conducted using a correlation metric and weighted average linkage clustering algorithm (WPGMA: Weighted Pair Group Method with Arithmetic Mean). Unlike other unsupervised machine learning segmentation techniques, hierarchical clustering does not require a user-defined number of clusters. The multi-level hierarchy allows the division of the 19 ROIs within the thalamocortical network into multiple groups based on the level that most accurately characterizes the dissimilarity between clusters. In the dendrogram, any two ROIs are eventually linked together at some level. The height of the links represents the cophenetic distance between two ROIs or group of ROIs (clusters). The cophenetic distance is a similarity measure that takes the gamma coefficients (amplitude and delay), regression coefficient (beta) and cycle-averaged PSC into account. Therefore, the shorter distance (vertical links) indicates closer similarity between the ROIs in the parametric space. The ROIs can be clustered into various groups/subnetworks based on user-defined threshold levels.

### Quantifying the similarity between networks under different stimulation conditions

We quantified the Dice similarity between hierarchically clustered networks for both the heat and heat + FUS stimulation conditions. The similarity between these clusters is gauged by the cophenetic distance, which is based on the temporal correlation between thalamocortical ROI pairs [e.g. primary somatosensory (SI)-VPL versus secondary somatosensory (SII)-VLP pairs]. After segmenting the ROIs into clusters, we calculated the inter-ROI (pair) correlation matrix, representing the correlation (*r* values) between ROI pairs. In the matrix plot, the arrangement of ROIs followed the classification observed during the heat stimulus condition. We defined valid overlap in our quantification by the difference in correlation ∇r = 0.15 between corresponding ROI pairs. To assess the suppressive effect of FUS at the thalamic nuclei, we evaluated the decrease in correlation power. This was defined by the cumulative squared correlation ($\sum r^{2})$ within the inter-ROI correlation matrix. Both the Dice similarity and the percentage change in correlation power were computed using Equations (1) and (2).


(1) ds=2×|X∩Y|/(|X|+|Y|)

(2) $$\nabla crP=\frac{\sum crH^{2}-crHF^{2}}{\sum crH^{2}}\times 100$$

Here, *X* and *Y* represent the set of clusters in the inter-ROI correlation matrix due to heat and FUS perturbation (Equation (1)). Similarly, *crH* and *crHF* denote the inter-ROI correlation coefficients associated with the corresponding clusters or subnetworks in both states (heat and heat + FUS, Equation (2)).

## Results

### EFC networks of thalamic VPL nucleus

**Figure 3:**
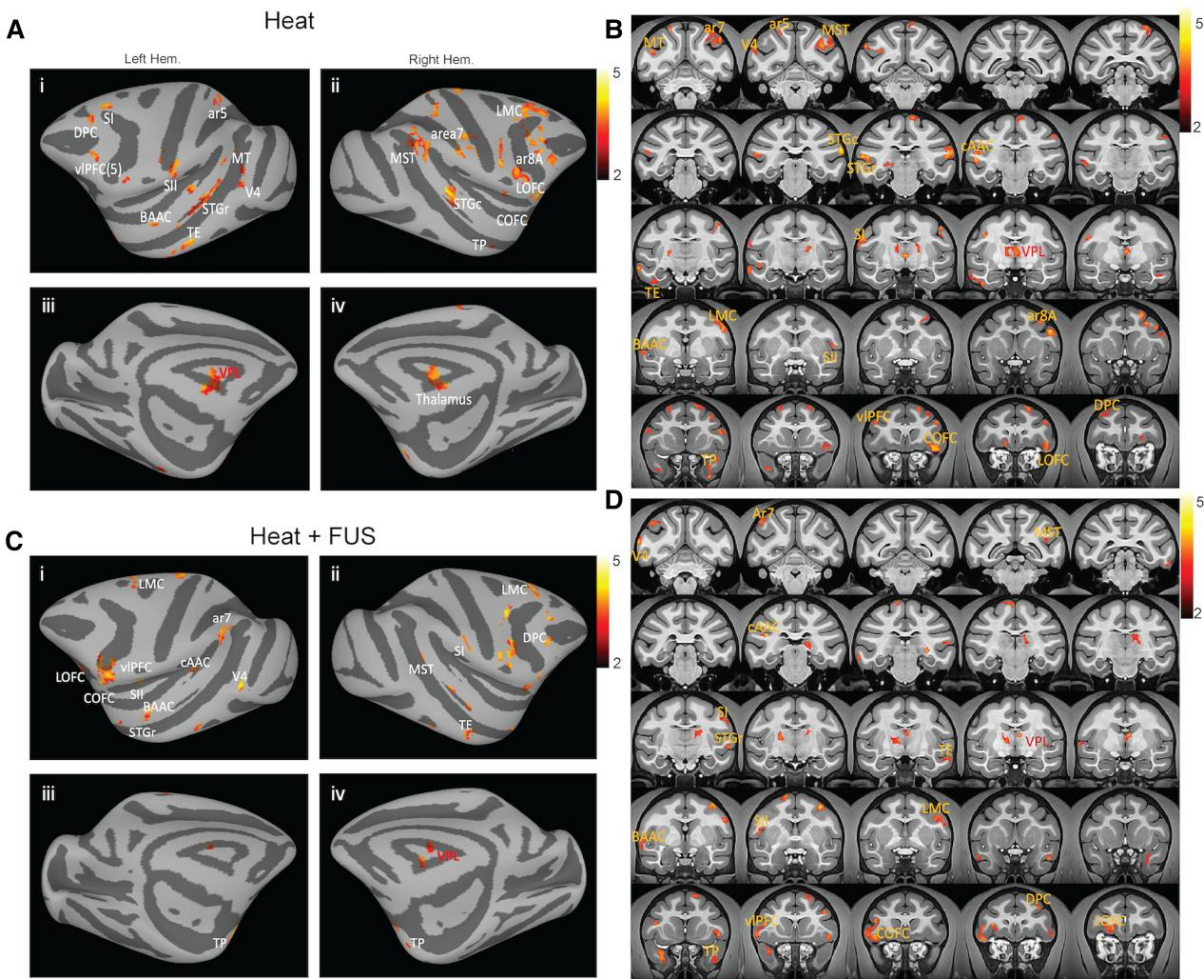
**EFC of thalamic VPL nucleus during heat versus heat + FSU stimulation.** Lateral and medial views of the EFC maps are overlaid on the inflated left (1, 3) and right (2, 4) hemispheres of the macaque brain during heat (**A**) and heat + FUS (**C**) stimulation. Posterior–anterior views of the EFC maps are overlaid on the coronal images for the heat (**B**) and heat + FUS (**D**) stimulation conditions. Colour bars indicate the correlation coefficient (*r*) values.

We first mapped the global EFC networks of the thalamic VPL nucleus involved in processing nociceptive heat stimuli and examined the effects of FUS modulation. Figure 3A and C depicts the lateral (1–2) and medial (3–4) views of the EFC maps of thalamic nuclei on an inflated macaque brain under heat (A) and heat + FUS (C) conditions. Figure 3B and D displays the corresponding thalamic EFC connections on coronal images (*t* > 2, *P* < 0.05, FDR corrected).

The thalamic VPL nucleus showed strong connectivity to 19 cortical areas during the processing of nociceptive heat stimuli. Table 1 shows the categorization of these areas by functionality. They encompass regions involved in processing somatic sensory dimensions (e.g. SI and SII areas, Area 5 and Area 7) and cognitive dimensions [e.g. lateral and caudal orbital frontal cortex (lat.OFC and caud.OFC), dorsolateral and ventrolateral prefrontal areas (DPC and vlPFC)] of nociceptive information (refer to Fig. 3A and B and Table 1). Notably, other sensory cortices, such as visual (V4, middle temporal (MT)) and medial superior temporal (MST) and auditory areas (Belt areas of auditory cortex (BAAC) and Core areas of auditory cortex (cAAC)), were also effectively connected with VPL, along with the primary and lateral motor cortex (M1/PM). The Brodmann Area 8 is equivalent to the frontal eye field in the human brain, which is a part of the frontal cortex and the dorsal attention network.^43-45^ Area 5 is part of the posterior parietal cortex and is involved in somatosensory processing, movement and association.^46^ The MST area is engaged in visual motion processing. Similarly, the temporal pole (TP) and inferotemporal cortex (area TE) belong to the temporal cortex and are involved in various functions such as visual pattern discrimination^47^ as well as multimodal sensory integration, memory and associations, emotion and social cognition and object and face recognition. Caudal superior temporal gyrus (STGc) and rostral superior temporal gyrus (STGr) are involved in various cognitive and perceptual functions. For example, STGr is involved in processing complex auditory stimuli and understanding the meaning of words and sentences and plays a role in recognizing and interpreting social cues. STGc houses the primary auditory cortex and is involved in processing speech sounds (known as Wernicke’s area), integrating auditory and visual information and perception of visual and auditory motion. In summary, various functions and properties of these areas have been extensively described in the literature, highlighting their intricate roles in sensory processing, attention, movement, emotion and cognition.

**Table 1.** Comparison of cortical areas exhibiting EFC to the right thalamic VPL nucleus under heat and heat + FUS conditions.

		Cortical subregions	EFC heat		EFC heat + FUS	
			L-hem	R-hem	L-hem	R-hem
Sub Net 1	1	Medial superior temporal area (MST)	0.0361	0.0324	0.0422	—
	2	TG temporal pole (TP)	0.0484	0.0365	—	0.0386
	3	Lateral orbital frontal cortex (lat.OFC)	0.0449	0.0362	0.0396	0.0314
	4	**Middle temporal area (MT)**	0.0382	0.0362	0.0324	—
	5	**Area8A (ar8A)**	0.0463	—	n	
	6	**Area 5 (ar5)**	—	0.0333	n	
	7	Primary somatosensory cortex (SI)	0.0265	0.0398	n	
	8	Rostral superior temporal region (STGr)	0.0375	0.0391	—	0.0364
	9	Belt areas of auditory cortex (BAAC)	0.0406	0.0423	0.0378	0.0362
	10	Ventrolateral prefrontal cortex (vlPFC)	0.0405	0.0389	0.0387	—
Sub Net 2	11	Caudal orbital frontal cortex (caud.OFC)	0.0354	0.0406	0.0395	0.0394
	12	Area7 (ar7)	—	0.0423	0.0355	0.0383
	13	TE area (areaTE)	—	0.0405	0.0399	—
	14	Visual Area 4 (V4)	0.0408	0.0407	0.0341	0.0403
	15	Core areas of auditory cortex (cAAC)	0.0413	—	0.0417	—
	16	Dorsolateral prefrontal cortex (DPC)	0.0497	—	0.0388	—
	17	M1/PM lateral motor cortex (LMC)	0.0315	0.0283	—	0.0375
	18	Secondary somatosensory cortex (SII)	0.0347	0.0382	0.0352	0.0379
	19	**Caudal superior temporal gyrus (STGc)**	—	0.0381	0.0421	—

Areas (bold) indicate regions that lost detectable EFC to VPL when the heat response was suppressed by concurrently delivered FUS. The mean *P*-value for voxels within the ROIs (*t* > 2, FDR corrected), derived from GLM analysis, is provided for each hemisphere (L: left; R: right). The letter “n” denotes brain regions that exhibited non-significant signal changes in both hemispheres, and ‘—’ refers to unilateral activation.

When the heat responses of the right thalamic VPL and surrounding nuclei were suppressed by FUS, the number of interconnected areas decreased from 19 to 15 (Table 1). Specifically, areas such as Area 8A, Area 5, the STGc, and the middle temporal (MT) area lost effective thalamic connections. Furthermore, we investigated the hemispheric asymmetry of the thalamocortical EFC networks. Given that the left hand was stimulated with heat, the right hemisphere was identified as the contralateral hemisphere. The results of statistical analysis (*P*-values) for each ROI in both hemispheres are detailed in Table 1, highlighting the differences between heat and heat + FUS conditions. The mean *P*-value was calculated for ROIs above the *t*-value threshold (*t* > 2, FDR, *P* < 0.05 corrected). A lower *P*-value therefore is associated with higher statistical significance of EFC between VPL and various ROIs.

These *P*-values for each ROI are one index of how reliable and statistically significant the EFC with VPL is for the ipsilateral and contralateral cortical ROIs. Out of the 19 areas examined, 13 ROIs in Table 1 displayed significant bilateral EFC under heat-only stimulation conditions. Four areas (namely caud.OFC, SII, Area 7 and Area 8A) showed significant EFC within the contralateral hemisphere, while a lack of EFC was observed between right VPL with the ipsilateral side of the brain. Across all ROIs, the effects of FUS can be characterized as: (i) no changes in EFC significance in both hemispheres, (ii) losing detectable EFC in both hemispheres and (iii) losing detectable EFC in the contralateral (right) or ipsilateral (left) hemisphere. For instance, the number of areas showing significant bilateral EFC decreased from 13 to 6, including caud.OFC, vlPFC, LMC, TP, area TE, and STGr and four areas—Area 8A, Area 5, STGc and MT—no longer had detectable EFC under heat + FUS stimulation. The loss of EFC in specific hemispheres underscores the profound effects of FUS modulation.

### Quantification of changes in thalamocortical EFC strengths

**Figure 4:**
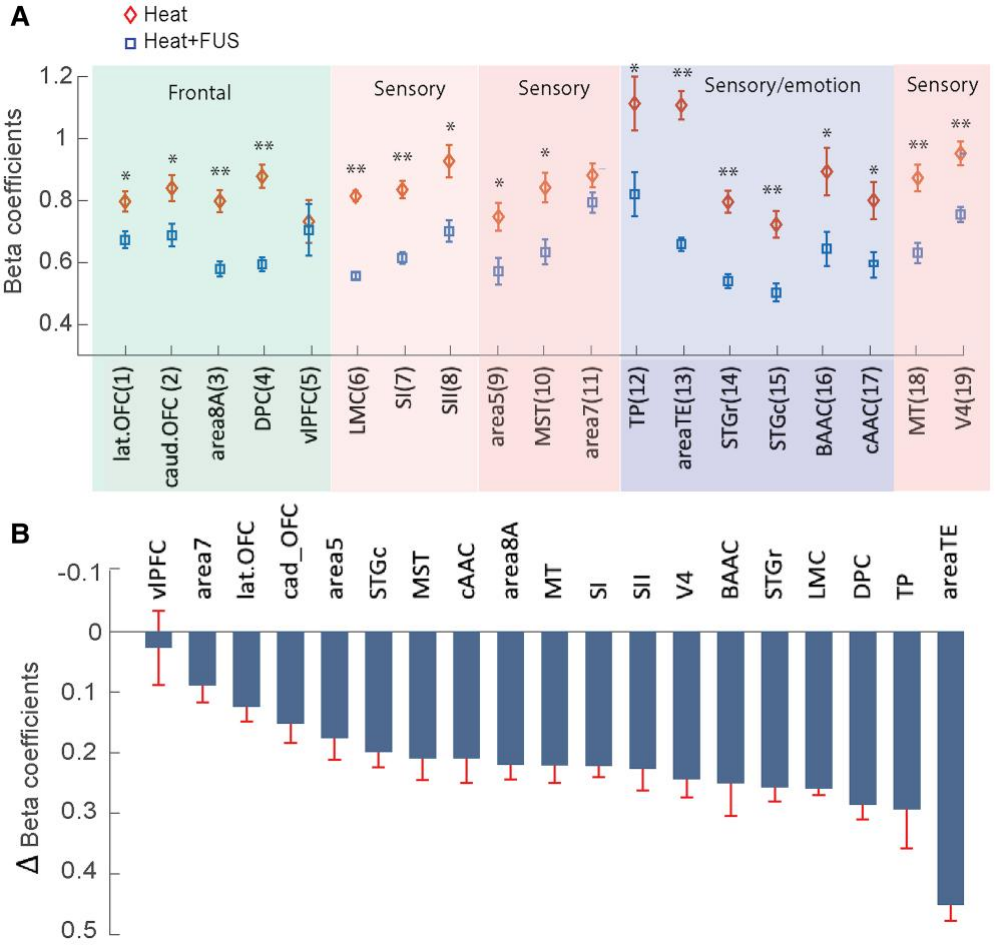
**Comparison of EFC strength under heat and heat + FUS conditions for each thalamocortical connection.** (**A**) The mean and standard error of the beta coefficient (derived from GLM analysis) for the heat (diamonds) and heat + FUS (squares) conditions. The 19 thalamocortical ROI pairs are ordered according to their functionality. (**B**) The reductions in beta coefficient values, obtained by subtracting heat values from heat + FUS values, are presented for each of the 19 thalamocortical ROI pairs (e.g. lat.OFC to V4), arranged in ascending order. The statistical significance of differences between the two stimulus conditions for each thalamocortical ROI was examined using a non-parametric MWW test (* *P* < 0.05, ** *P* < 0.005, *n* = 24).

We further evaluated the effects of FUS on thalamocortical EFC strength for each area using beta coefficients (Fig. 4). These beta values indicate the effective connectivity strength of each ROI to the thalamic VPL in heat and heat + FUS conditions. We found that FUS suppression of thalamic nuclei significantly reduced connectivity strengths in 19 ROIs, with the exception of Area 7 and vlPFC, where the changes were statistically insignificant (Fig. 4A). The changes in effective connectivity between the two conditions (heat and heat + FUS) were further illustrated by the differences between the beta coefficients of the two conditions, presented in ascending order according to the extent of difference (Fig. 4B). This differential beta value serves as a direct indicator of either an excitatory or inhibitory effect of FUS. Notably, primary somatosensory and other high-order sensory areas (SI, SII, V4, MT, STG, LMC, and BAAC) exhibited reductions in beta coefficients of ∼0.2, whereas cognitive areas such as the vlPFC, lateral OFC and caudal OFC showed relatively smaller reductions. In contrast, high-order cognitive and emotional processing areas, including DPC and TP, displayed robust reductions in connectivity, with area TE showing the strongest reduction, nearing 0.5.

### Hierarchical organization of thalamocortical EFC and changes with FUS thalamic suppression

**Figure 5:**
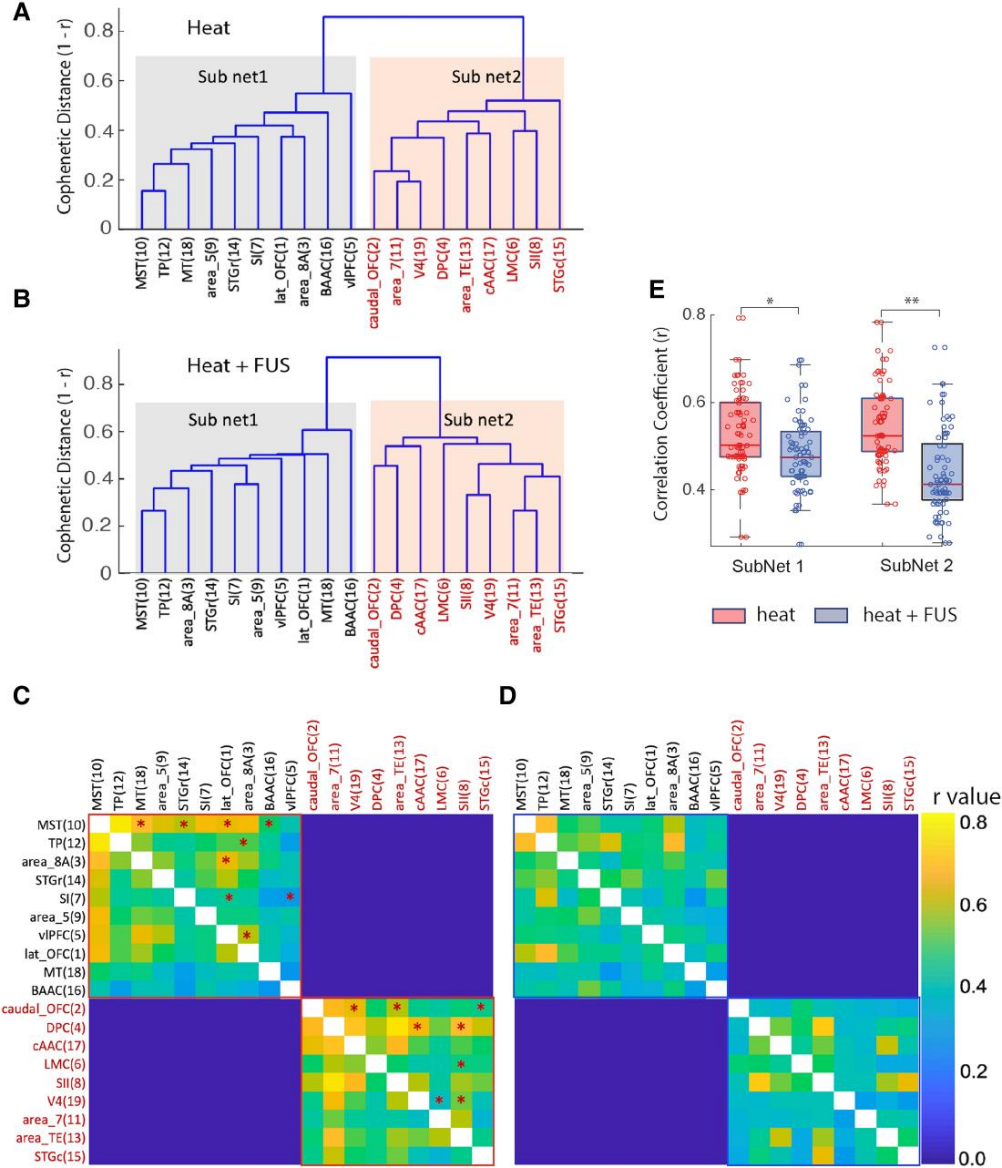
**Comparison of hierarchical clustering organizations of thalamocortical EFC networks under heat and heat + FUS stimulation conditions.** (**A**) and (**B**) Dendrogram tree plots display the hierarchical cluster organization for 19 thalamocortical area pairs (as outlined in Table 1). The *y*-axis represents the cophenetic distance (*d* = 1 − *r*) between ROIs, which is an inverse measure of the correlation between mean time course of ROI pairs in the template space. ROIs are grouped into clusters and subnetworks using the WPGMA algorithm. Matrix plots present mean correlation coefficient between ROI pairs in each run for heat (**C**) and heat + FUS (**D**) stimulation conditions. Asterisk */** indicates statistically significant differences (*/**, *P* < 0.05/0.005, FDR corrected) of inter-ROI correlation between the two conditions for each ROI pair, as determined by the MWW test. The scale bar represents correlation coefficient *r* values. (**E**) The Whisker boxplots illustrate the upper, lower quartile and median value (horizontal red bar). The distributed circles refer to the mean inter-ROI correlation in the Subnetworks 1 and 2. Each circle represents element of the subnetworks in both states (heat and heat + FUS, marked by boundary in **C** and **D**. The asterisk */** on top of horizontal bars indicates statistically significant differences (*/**, *P* < 0.05/0.005), as determined by the MWW test. Each data point in matrix plots represents mean inter-ROI correlation averaged across 24 runs acquired from four animals (animal #1: 6 runs, animal #2: 4 runs, animal #3: 6 runs and animal #4: 8 runs).

Two different correlation measures were quantified and presented in Fig. 5. Figure 5A and B presents dendrograms depicting the hierarchical cluster organization of 19 regions within the nociceptive thalamocortical networks under heat (Fig. 5A) and heat + FUS (Fig. 5B) stimulation conditions. The *y*-axis represents cophenetic distance (*d* = 1 − *r*), evaluated using the WPGMA algorithm, a correlation-based distance measure. This distance is an inverse function of the correlation between the parameterized input vectors, which incorporate multiple parameters, including amplitude and delay of the two-gamma fit, beta coefficient and PSC, for hierarchical clustering. Essentially, a larger distance between ROIs or clusters suggests less correlated BOLD time courses or poor similarity in EFC. When the cophenetic distance was set at 0.7 on the dendrogram, the thalamocortical EFC networks branched into two subnetworks (Sub Net 1—blue and Sub Net 2—red) under both stimulus conditions. Under heat stimulation, in Sub Net 1 (Fig. 5A, blue), the thalamic EFC with MST and TP showed a strong correlation, grouping them together (*d* = 0.17). These two areas formed another distinct cluster (*d* ∼ 0.28) with MT. Area 5, STGr and SI clustered together at a larger distance (*d* ∼ 0.38). The connectivity of vlPFC with the rest of the members in Sub Net1 1 at a larger distance (*d* = 0.6) indicates a weak correlation with the other areas. In Sub Net 2 (Fig. 5A, red), a strong correlation between Area 7 and V4 was noticeable (*d* = 0.2). Around the *d* = 0.4 level, caudal OFC, DPC, area TE and cAAC were clustered together, as did LMC and SII. STGc similarly exhibited a larger distance, indicating weak correlations with the rest of the members in Subnetwork 2.

The concurrent delivery of suppressive FUS to the right thalamic VPL nucleus led to distinct changes in the organization of clustered ROIs. Although the ROIs belonging to the two primary subnetworks remained unchanged, there was an overall reduction in inter-ROI correlation, as seen by ROIs connecting with each other at comparatively larger distances (*d* > 0.4, Fig. 5B). For example, within the blue Sub Net 1 1, the cluster formed by MST, TP and Area 8A remained largely unaltered, although inter-ROI correlations were reduced. Similarly, within the red Sub Net 2, the overall distances between ROIs increased, and the organization of the clustered members was also altered. However, the strongly correlated pair (Area 7 and V4) under heat stimulation underwent significant changes, showing weaker connectivity when FUS was added to the heat stimulation. Improved connectivity, indicated by smaller d values, was observed between V4 and SII. Notably, SII shifted its close connection from LMC during heat stimulation to V4 during heat + FUS condition. Lastly, cAAC and LMC exhibited weakened connectivity to the other areas. The change in organization of the networks describes the association/dissociation of areas with each other at various levels, cophenetic correlation coefficient measures how faithfully the dendrogram can separate the clusters.

The cophenetic correlations for two networks were 0.68 and 0.72, respectively, which indicates FUS increases the correlation between areas within the subnetworks. The cophenetic correlation measures how closely the ROIs within each subnetwork relate to the overall distance between the subnetworks in each condition (heat versus heat + FUS). A relatively smaller separation (distance) between subnetworks compared with inter-ROI correlation (within the subnetwork) can increase the cophenetic correlation value, even if there is an overall reduction in inter-ROI correlation when FUS is added to the heat stimulus (heat + FUS). Therefore, the cophenetic coefficient reflects clustering efficacy within each condition but may not serve as an absolute measure of overall changes in inter-ROI correlation. The statistical difference between the cophenetic distances measured using MWW test was also significant (*P* < 0.001).

### Effects of FUS on inter-ROI correlation coefficients

To understand the effects of FUS-induced thalamic suppression on thalamocortical EFC networks, we computed correlation coefficients (thresholded at *r* > 0.3) between all thalamocortical ROI pairs and presented them in matrix plots. Unlike the hierarchical clustering analysis, which uses parameterized input vectors as additional shape parameters, this correlation is calculated based on the temporal dynamics of the mean time course. A high *r* value between two ROI pairs indicates that these two ROI exhibited highly correlated changes in their EFC strength to the thalamic VPL nucleus when FUS suppression was applied. Figure 5C depicts strong correlations between many ROIs within the red or blue subnetworks (as indicated by hot ‘colour’ patches), closely resembling the cophenetic distance on an inverse scale. When FUS modulation was applied, the correlation strengths between ROIs changed significantly (Fig. 5D). Compared with the heat-only condition, FUS suppression of thalamic heat response resulted in weakened EFC for most regions and strengthened EFC in few of them (Fig. 5C and D). The red asterisks on each ROI pair indicate statistically significant differences (*P* < 0.005, FDR corrected) between the heat and heat + FUS conditions. We also conducted a non-parametric MWW tests performed on data derived from different samples (number of animals = 4, 24 runs in total) and observed significant reductions in connectivity strength (*r* values) in both subnetworks (Fig. 5D).

The Dice similarity of the EFC network (functional connectivity matrix, Fig. 5C and D) between heat versus heat + FUS conditions was calculated to be 0.7053. The differences from 1 can be attributed to the application of FUS. This calculation used a differential correlation ∇r = 0.15 between corresponding inter-ROI pairs as the similarity criterion. The percentage decrease in correlation power due to FUS (Equation (2)), measured using the squared cumulative difference in correlation, was estimated to be 17.71%. This aligns closely with the acceptable criterion (∇r = 0.15) for overlap when assessing Dice similarity. The reduced Dice coefficient reflects the dissimilarity between the two subnetworks under the given similarity criterion ∇r, and the overall reduction in inter-ROI correlation power ($\sum r^{2}$) indicates weaker connectivity among the ROIs pairs due to the application of FUS.

### Effects of FUS on EFC strength of each ROI within each subnetwork

**Figure 6:**
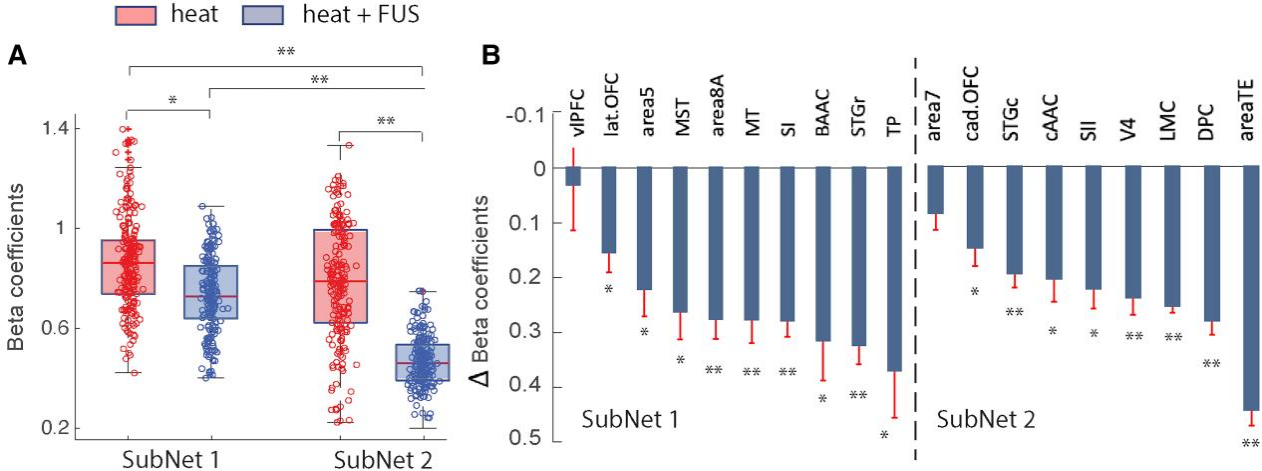
**FUS effect on EFC subnetworks.** (**A**) Boxplots show the median (indicated by horizontal red bars) and distribution of inter-ROI beta coefficients of EFC for all ROIs within each subnetwork (Sub Net 1 and Sub Net 2) during heat (red) and heat + FUS (blue) stimulation. Each circle represents the measured beta coefficients associated with each ROI in the subnetworks (19(10 + 9) ROIs in two subnetworks) for each run (*n* = 24 runs, animal #1: 6 runs, animal #2: 4 runs, animal #3: 6 runs and animal #4: 8 runs). The asterisk */** on top of horizontal bas indicates statistically significant differences (*/**, *P* < 0.05/0.005), as determined by the MWW test. (**B**) Bar plots represent the mean magnitude and standard error of reductions in beta coefficients of EFC for each ROI within each subnetwork, arranged in ascending order of reduction.

Using hierarchical clustering analysis, we identified two subnetworks and examined the effects of FUS on the strengths of thalamocortical EFC within each subnetwork, as measured by the magnitude of the beta coefficient for each ROI and collectively for the subnetworks. Each measurement represents the inter-ROI pair in a data set, and the statistical significance of difference was assessed using the MWW test performed on data derived from different samples (number of animals = 4, 24 runs in total). The boxplots in Fig. 6A show a significant reduction in thalamocortical EFC strength across all ROIs within each subnetwork when FUS suppressed the thalamic VPL heat responses. Figure 6B illustrates the magnitude of reduction caused by FUS across ROIs within each subnetwork. Notably, sensory areas such as SI, SII and Area 5 exhibited a substantial reduction in connectivity strength to the thalamic VPL nucleus. The least affected areas were vlPFC for Sub Net 1 and Area 7 for Sub Net 2. The effects of FUS suppression on thalamocortical EFC are particularly significant for ROI pairs between VPL-TP in Sub Net 1 and VPL-area TE in Sub Net 2.

### Schematic summary of the nociceptive thalamocortical EFC reorganization caused by FUS-induced thalamic suppression

**Figure 7:**
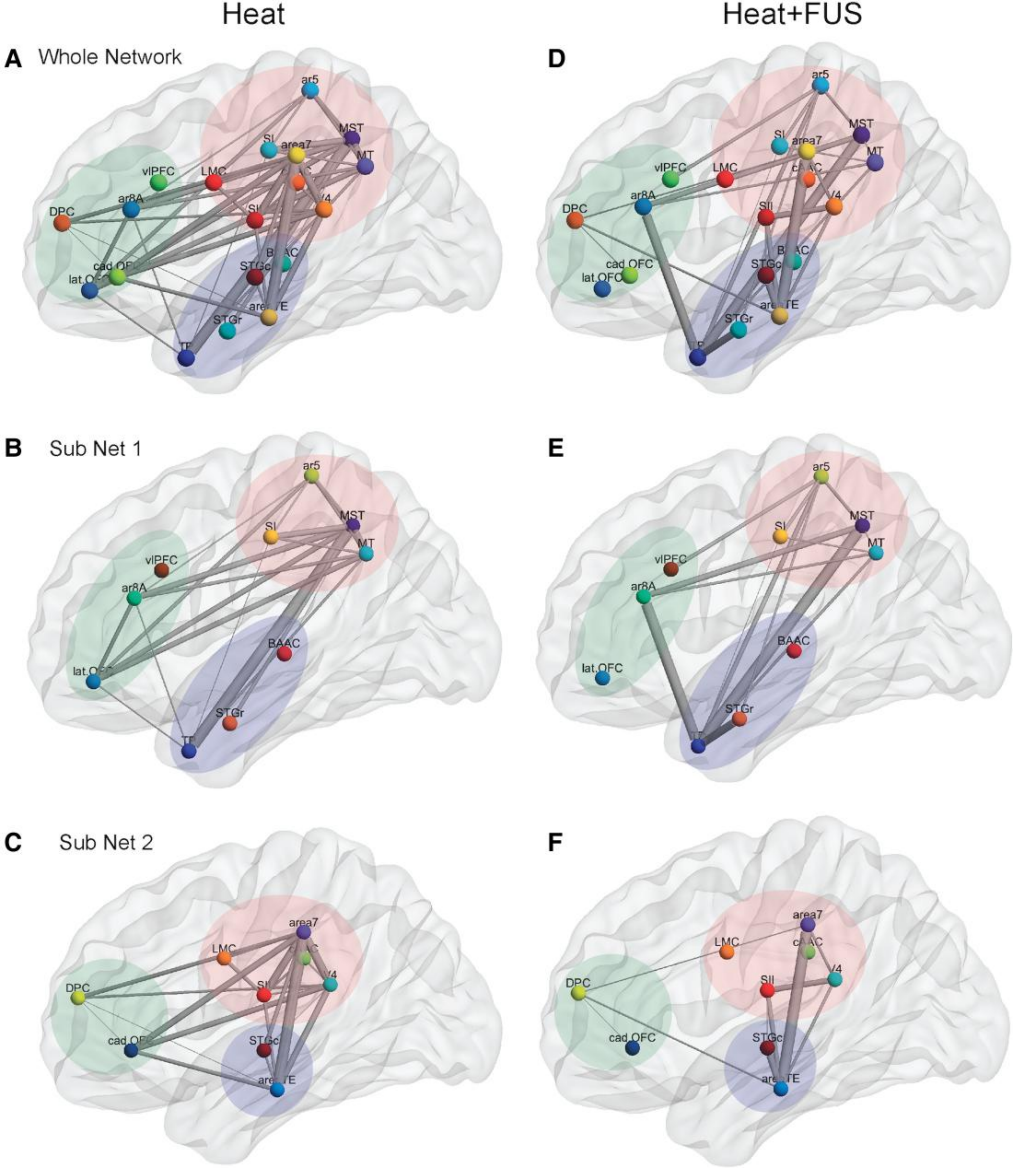
**Graphical presentation of FUS-induced EFC network reorganization.** Labelled and colour-coded nodes represent the whole-brain ROI pairs grouped with hierarchical clustering for the heat (**A**) and heat + FUS (**D**) conditions. Functional organization of EFC within Subnetworks 1 (**B** and **E**) and 2 (**C** and **F**) is shown for the heat (**B** and **C**) and heat + FUS (**E** and **F**) stimulation conditions. The weighted inter-nodal lines represent the inter-ROI correlation coefficients normalized to values above *r* > 0.5 based on the data used for group analysis in the hierarchical clustering (24 runs acquired using four animals, *n* = 4).

We summarize and illustrate the FUS-induced changes in nociceptive thalamocortical EFC networks using graphic glass brain presentations (Fig. 7). The whole-brain network and the two subnetworks are plotted separately, highlighting several changes in EFC network organization. First, the thalamic VPL connects to many cortical areas from different functional networks during thermal nociceptive information processing. Second, suppressing the nociceptive response of the thalamic VPL leads to a brain-wide reduction in inter-ROI pair connection strengths, as seen when comparing Fig. 7A–C with 7D–F. Third, a few ROI pairs, including Area 8A and TP, TP and STGr, STGc and Area TE, and SII and V4, along with vlPFC and Area 5, exhibited strengthened EFC. Both the weakening and strengthening of EFC were not confined to sensory circuits and were observed across multiple brain networks. In conclusion, the suppression of nociceptive heat response in the thalamic VPL and surrounding nuclei profoundly reorganized the thalamic EFC networks. This reorganization is brain-wide and involves cortical regions mediating sensory, motor, emotional and cognitive functions.

## Discussion

The thalamus serves as a central relay station for nociceptive processing and is essential to pain perception. Among subcortical regions, the VPL and MD nuclei are the most consistently implicated in pain processing, showing reliable activation in human fMRI pain studies.^48^ The VPL nucleus receives input primarily from the STT, which carries pain and temperature information from the body, and relays this information to the somatosensory cortices for sensory processing and integration. The ventrocaudal nucleus, a homologous structure to the VPL (including the VPM) in the human brain, has been targeted for pain relief through DBS. However, there are mixed reports on the clinical benefits of this DBS procedure, as reviewed by Boccard *et al.*^49^ and Frizon *et al*.^50^ A recent ultrasound stimulation study showed that stimulating the anterior thalamus altered the subjects’ pain detection thresholds.^16^ To our knowledge, no studies have explored the impacts of thalamic modulation on nociception networks and information processing. Here, we present the first report on the targeted FUS suppression of the thalamic VPL nucleus and its impact on the organization of the effective thalamocortical nociceptive network.

### Low-intensity FUS is an effective neuromodulation tool

Numerous studies have demonstrated that transcranial low-intensity FUS is an effective neuromodulator capable of altering behaviour, neuronal electrical activity and BOLD signal changes in both human subjects/patients and animal models.^9,51,52^ The reduction of evoked potential following FUS application has been well documented in rodents, rabbits^53-55^ and humans.^56^ Although both excitatory and inhibitory effects of FUS have been observed with varying pulse frequencies in the 220–250-kHz range, the exact mechanisms underlying these neuromodulation-induced BOLD signal changes remain elusive.^57^ Theoretical modelling of biological effects predicts that FUS can inhibit neurons in the mouse and human somatosensory cortex^56,58^ and has the potential for both excitation and inhibition^59^ of neural activity.

In our observations, both activation and inhibitory effects of medium-amplitude transcranial FUS were evident.^8,9^ When tactile stimuli were combined with FUS targeting Areas 3a and 3b, we noted a suppression in the BOLD response. A quantitative evaluation showed that the inhibitory effect of FUS was also evident in terms of PSC throughout the brain, particularly in the Areas 1 and 2, SII, MCC, VPL and insula^7,9^ In the data analysis for heat stimulation, both excitatory and inhibitory effects of FUS were observed using GLM. However, the inhibitory effect was more prominent in the effective connectivity modelling of EFC for all 16 ROIs identified in the heat + FUS condition. Our observations of profound alterations in thalamocortical EFC induced by FUS underscore the therapeutic potential for FUS in pain management.

### Hierarchical organization of thalamocortical EFC networks of VPL

Hierarchical clustering delineated two main subnetworks within the VPL-cortical network. VPL was connected not only to sensorimotor and associated cortical areas such as SI, SII, LMC (lateral motor cortex; M1/PM), Area 5 and Area 7, but VPL also connected to a broader range of cortical areas (outlined in Table 1), including higher-order cognitive areas such as vlPFC, DPC, lat.OFC, caud.PFC and Area 8 during nociceptive processing. Interestingly, the two somatosensory cortices—SI and SII—both extensively interconnected to VPL, were clustered into two separate subnetworks. The areas strongly connected to VPL by EFC were not the SI and SII cortices but rather associative and higher-order regions involved in emotional and cognitive functions.

For example, within the SI subnetwork (Sub Net 1), thalamic connections to MST, TP, lat.OFC and MT were notably strong. Three of these four areas play important roles in visual motion and depth perception (MST and MT) and multisensory processing (TP). The lat.OFC is engaged in higher cognitive functions and emotional processing, including inhibitory control, decision-making and emotional regulation. SI and Area 5 were clustered together and had comparable connectivity strength to the thalamic VPL. These areas are associated with sensory processing and integration. Area 8, part of the frontal eye field, plays a role in higher cognitive functions such as decision-making—especially in tasks requiring the suppression of automatic responses, attention and working memory. The auditory cortex (BAAC) and vlPFC were loosely interconnected.

Within the SII (Sub Net 2), SII is strongly interconnected with LMC (M1/PM), while Area 7, Area TE and V4 (or inferotemporal cortex) are also strongly linked. These regions play significant roles in higher-order sensorimotor information processing and integration, including nociception (Area 7 and SII), vision (V4), motor planning and execution (LMC) and object recognition (area TE). The caudal orbital frontal cortex (caud.OFC) is clustered closely with these areas (Fig. 4A) and is involved in sensory integration, reward and punishment evaluation, impulse control and emotion processing. Superior temporal gyrus (STGc), dorsolateral frontal cortex (DFC) and cAAC were relatively loosely connected. The caudal (posterior) portion of the superior temporal gyrus (STGc) is important for multisensory integration and auditory processing, while DFC plays a critical role in higher cognitive functions, including executive control, emotional regulation and attention.

VPL’s thalamocortical connections to somatosensory (SI, SII) and associative (Area 5 and Area 7) regions are expected during nociceptive processing due to their known anatomical connections. However, VPL’s connections to visual, auditory and motor areas (LMC) are somewhat unexpected. Its connection to higher-order areas involved in complex and/or multisensory integration, such as STG, MST and TP, highlights the diverse connections of VPL. Of particular interest is VPL’s connection to higher cognitive areas, such as PFC and DFC, during nociceptive processing, even under anaesthesia. This finding suggests that VPL communicates with higher cognitive areas during nociception without consciousness. The role of these higher-order areas in nociception warrants further investigation.

### FUS thalamic suppression induced network-wise reorganization

Suppressing the thalamic VPL heat nociceptive response enables the assessment of its excitatory or inhibitory effective connections to cortical areas. Areas that show strengthened EFC connections during FUS-induced suppression likely indicate a suppressive control exerted by the thalamic VPL. In contrast, areas with weakened connectivity suggest direct and causal connections to the thalamic VPL, where a decrease in VPL response leads to reduced effective connections with these areas. Hierarchical clustering demonstrated that changes in EFC connection strength and reorganization occurred primarily within subnetworks. Suppression of VPL predominantly induced a reduction in subnetwork-wise connection strength (as indicated by reduced Beta coefficients in Fig. 5) and decreased the number of connections between thalamocortical area pairs (as shown by reduced edge density in Fig. 6). Despite these changes, the overall subnetwork structures remain intact. Among the 20 areas studied, only Area 7 did not display significant connection strength to VPL during FUS. The universal reduction in connectivity across the other 19 areas suggests their direct interconnection with thalamic VPL. A decrease in the VPL heat response corresponds to a reduced correlation with these areas.

Within each subnetwork, significant alterations in inter-area connections occur in all ROI pairs when the thalamic heat response is suppressed by FUS. For instance, within the SI subnetwork, sensory and sensory-associated areas undergo reorganization but remain closely clustered. The most notable change is in the vlPFC, where its previously weak connections to other areas strengthen, particularly with Area 5. The removal of the thalamic heat response at VPL strengthens the link between vlPFC and Area 5, suggesting that Area 5’s connection to vlPFC was previously suppressed by VPL during heat processing. Within the SII subnetwork, significant changes in cluster structures are observed in the auditory cAAC and motor LMC regions, which display weaker clustering with other areas. These observations suggest that VPL modulation has the most pronounced effects on higher cognitive, auditory and motor areas.

While statistical significance is crucial for confidently describing alterations in inter-ROI correlations, objective parcellation across various hierarchical levels can elucidate neuromodulation through changes in cluster organization within the subnetwork. Unlike other unsupervised learning algorithms, hierarchical clustering segregates data without predefined segment numbers, organizing it into a multi-level hierarchy where all clusters and members connect at specific levels in the parametric space. This approach allows for quantifying dissimilarity and overlap between specific subregion levels. The Dice similarity measure further substantiated these organizational shifts during FUS suppression.

### MRI-guided FUS—a promising tool for device-based pain relief

Current approaches to clinical pain management primarily fall into two categories: pharmacological or device-based therapies. Device-based pain therapies often involve either surgical interventions (decompressive or ablation) or devices that target specific parts of the nervous system. These therapies, including ablative procedures and neuromodulation, are important alternatives to drugs and do not carry the risk of addictive side effects. Compared with irreversible ablative procedures, neuromodulation is testable and reversible.

Current neuromodulation tools used in pain clinics include spinal cord stimulation, transcutaneous electrical nerve stimulation, transcranial magnetic stimulation (TMS), transcranial direct current stimulation (tTDS), as well as DBS.^1,2^ While DBS offers high targeting precision, it requires a highly invasive procedure involving the surgical implantation of electrodes into the brain targets. Non-invasive techniques like repeated rTMS^3,4^ or tDCS^5,6^ also show promise, though their therapeutic benefits vary across patients and different chronic pain disorders.^7-9^ These variable clinical outcomes have been attributed to the functional heterogeneity of brain targets among patients, the lack of objective measures to evaluate treatment effects and a limited mechanistic understanding of how neuromodulation affects targets and beyond.

Among the existing neuromodulation approaches, DBS of the thalamic sensory nuclei, periventricular grey/periaqueductal grey and recently anterior cingulate cortex (ACC), using implanted electrodes, has shown the most promise for relieving pain or improving quality of life.^2,10-14^ However, the ability to screen for the potential DBS effects or to identify other targets remains extremely limited. In this context, non-invasive MRgFUS offers the advantage of simultaneously monitoring target engagements and observing stimulation effects beyond the immediate target. This capability provides valuable information for understanding therapeutic effects and optimizing target selection, neuromodulation protocols and dosing. This study presents the first line of evidence supporting the effectiveness of FUS in modulating thalamic nociceptive activity and thalamocortical networks, providing preclinical data for future clinical trials aimed at pain relief using FUS.

### Advantages of MRgFUS neuromodulation for future human clinical trials

The successful development of an MRgFUS system has the potential to revolutionize the treatment of various pain disorders, offering capabilities that have not been previously achievable. Specifically, MRgFUS can be used not only to perform personalized DBS targeting and assist in selecting responders for invasive procedures like DBS but also to non-invasively and reversibly test the effects of transient neuromodulation on different hubs within the pain circuit. This approach provides an efficient method for identifying new therapeutic targets for pain treatment and offers unprecedented opportunities to gain a conclusive mechanistic understanding of various chronic pain disorders.

The ability to link neuromodulation of specific targets and subjective pain experiences will facilitate the testing of the treatment efficacy for both existing and new methods, guiding the development of individualized therapies in the future. To date, MRgFUS is the only method capable of delivering non-invasive neuromodulation with high spatial precision and functional feedback. This capability is essential for future clinical trials, especially as breakthroughs in functional imaging studies of chronic pain disorders have shifted the traditional view of pain perception towards a pain neurometric theory. This theory emphasizes the collective role of multiple brain regions and networks in complex chronic pain experiences. A better understanding of this neural circuity will be critical to addressing the differential success associated with various techniques, and the insights gained will inform potential synergistic opportunities. A fully developed and integrated MRI-guided FUS neuromodulation system—featuring simultaneous functional feedback, high target precision, therapy reversibility and flexibility in personalized target selection—would offer capabilities unmatched by any other current method.

### Accuracy of MRI-guided FUS modulation and technical considerations

Accurate FUS target engagement is crucial for inducting effective neuromodulation. The FUS beam interacts differently with cortical areas compared with deep brain regions due to their distinct structural and functional organizational characteristics. Deep brain regions, such as the thalamus, are typically densely packed with neurons and located near one another. As depicted in Fig. 2, thalamic targeting was informed by pre-acquired fMRI activation maps. However, despite these efforts, the actual thalamic nuclei influenced by the FUS beam exhibited some variability. This inconsistency undoubtedly can affect the overall neuromodulation outcomes. To address this variability, a system that enables high-precision targeting, provides real-time confirmation of target engagement (via fMRI or MR-ARFI) and offers instant reference to an atlas or probability maps for specific brain regions of interest would be invaluable. Such a system would be beneficial not only for preclinical and clinical research but also for patient treatments. Future efforts should focus on incorporating these features into clinical FUS systems to enhance accuracy and effectiveness.

### NHP brain as a model system for preclinical pain research

Both acute pain and chronic pain are mediated by coordinated activity across a wide range of brain regions and networks. fMRI studies on human pain networks have delineated a complex pain matrix consisting of brain regions and networks that extend beyond those involved in processing and encoding sensory pain experiences.^1-3^ Prior studies by our team and others have shown that many of these putative pain regions, identified in conscious human subjects and patients, also exhibit significant fMRI signal changes under anaesthesia in NHP brains (macaques and squirrel monkeys) during the processing of various nociceptive inputs..^4-6,17^ These regions, referred to as the ‘nociception matrix,’ retain their involvement even under anaesthesia, underscoring their fundamental roles in processing and integrating nociceptive information. This makes them highly suitable models for further exploration of their causal interconnections using neuromodulation tools that are not readily available for use in humans.^7-12^

## Supplementary Material

fcaf062_Supplementary_Data

## Data Availability

The data, documentation and code that support the findings of this study will be made available in the publicly available repository: http://openneuro.org.
