# Supplementary material for: Modulating nociception networks: the impact of low-intensity focused ultrasound on thalamocortical connectivity
Source: Brain Commun. 2025 Feb 8;7(1):fcaf062. doi: 10.1093/braincomms/fcaf062 (PMC11878384; doi:10.1093/braincomms/fcaf062)
Supplement: fcaf062_Supplementary_Data [file fcaf062_supplementary_data.pdf]

Supplementary Material

Supplementary Table 1. Transducer and Drive System Parameters

	Manufacturer	*Model Number*	*Center Frequency*	*Radius of Curvature*	*Diameter*	*Number of Elements*	*Element Distribution*
Transducer	Imasonic	Custom Design	650 kHz	72 mm	103 mm	128	randomized array of 6.6 mm elements on a spherical surface
Matching	Custom Design with Image Guided Therapy Guidance						
Integrated drive system	Image Guided Therapy						

Supplementary Table 2. Pulse Timing Parameters used During Experiments

	Duration	*Ramp Duration*	*Ramp Shape*	*Repetition Interval*
Pulse	500 us	50 us	Linear	1 ms
Pulse train	500 ms	-	-	2 sec
Pulse train repeat	16 sec	-	-	Minimum of 46 sec

Supplementary Table 3. Free Field Pressure Parameters

Experiment	P_{sp} *(MPa)*	*MI (no de-rating)*	*Steering Coordinates [X, Y, Z] (mm)*	*Axial Position of* P_{sp} *[X, Y, Z] (mm)*	*Centroid of -3dB pressure region [X, Y, Z] (mm)*	*Axial Focus Size (-3dB) (mm)*	*Lateral Focus Size (-3dB) (mm)*	*Axial Focus Size (-6dB) (mm)*	*Lateral Focus Size (-6dB) (mm)*
1	1.4	1.74	[-3.4, 3.1, -7]	[-3.25, 3, -6.25]	[-3.28, 3.01, -6.57]	7.32	1.66	10.01	2.41
2	1.4	1.74	[-2, 2, 0]	[-2, 2, 0.25]	[-1.96, 1.96, 0.14]	6.24	1.58	9.32	2.22
3	1.4	1.74	[-6.3, -1, 0]	[-6, -1, 0.25]	[-6.16, -0.95, 0.14]	6.83	1.73	10.02	2.55
4	1.96	2.43	[0, 0, -5]	[0, 0, -4.5]	[-0.013, 0.0067, -4.67]	6.71	1.57	9.56	2.17
5	1.68	2.08	[0, 0, -5]	[0, 0, -4.5]	[-0.013, 0.0067, -4.67]	6.71	1.57	9.56	2.17

Notes: Reference plane is coincident with the position of the maximum of the focus generated during an unphased transmission. Measurements at specific steered locations acquired through free-field simulations and agree closely with hydrophone measurements made on a regular grid.

Supplementary Table 4. Estimated Transcranial Pressure Parameters Based on Simulations

Experiment	P_{sp} *(MPa)*	*MI (derated according to simulation)*	*Steering Coordinates [X, Y, Z] (mm)*	*Axial Position of* P_{sp} *[X, Y, Z] (mm)*	*Centroid of -3dB pressure region [X, Y, Z] (mm)*	*Axial Focus Size (-3dB) (mm)*	*Lateral Focus Size (-3dB) (mm)*	*Axial Focus Size (-6dB) (mm)*	*Lateral Focus Size (-6dB) (mm)*
1	0.76	0.94	[-3.4, 3.1, -7]	[-3.75, 3, -7.5]	[-3.92, 2.99, -6.92]	6.38	1.86	8.28	2.71
2	0.9	1.12	[-2, 2, 0]	[-2, 1.75, 0]	[-2.02, 1.81, -0.22]	6.67	1.61	10.06	2.35
3	0.9	1.12	[-6.3, -1, 0]	[-6, -1, 0.25]	[-5.98, -1.02, 0.28]	7.15	1.84	10.74	2.73
4	0.86	1.07	[0, 0, -5]	[-0.5 -0.25, -6]	[-0.74, -0.68, -5.04]	9.68	2.11	10.96	3.1
5	0.85	1.05	[0, 0, -5]	[-0.25, 0, -5.75]	[-0.31, -0.04 -5.95]	7.04	1.69	10.08	2.57

Notes: Reference plane is coincident with the position of the maximum of the focus generated during an unphased transmission Measurements acquired through simulations.

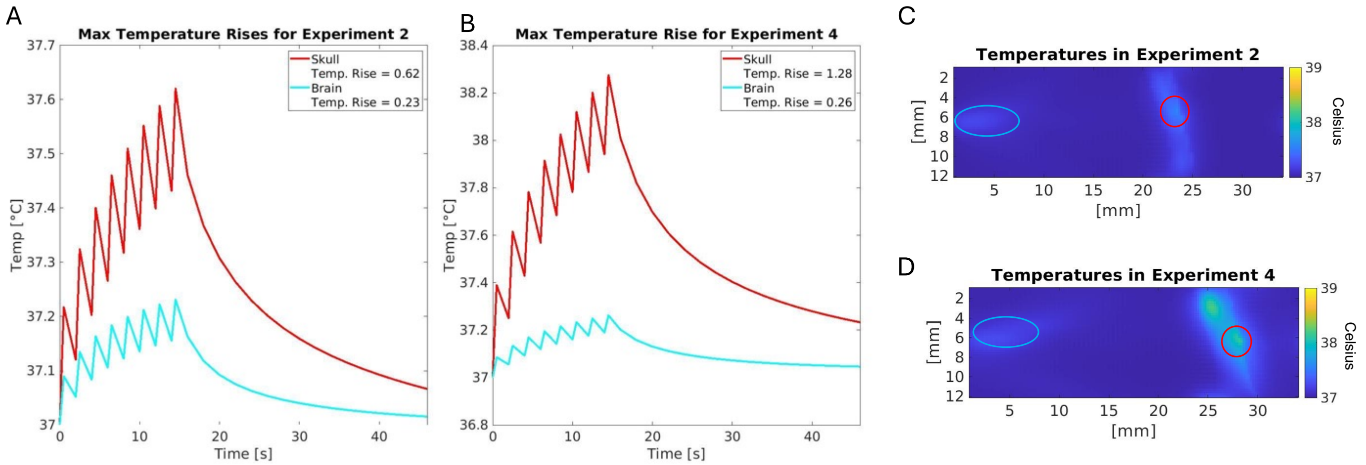

Supplementary Figure 1. Temperature simulations. Simulations (N = 2) of the highest estimated pressure in the brain (A) and the highest transmit power used (B) show a maximum temperature rise of approximately 0.6 C and 1.3 C, respectively in the skull, while the brain temperature increased by 0.23-0.26 C. A map of the simulated thermal rise during the highest estimated pressure (C) and highest transmit power (D) show the 2D distribution of temperature with circles outlining the location of the maximum (red) and focal (blue) temperature.

Supplementary Table 5. Parameters used for thermal simulations.

Parameter	Medium	Value	Units
<u>Thermal</u>			
Specific heat	Skull	1700	[J/(kg*K)]
Specific heat	Water	3600	[J/(kg*K)]
Thermal conductivity	Skull	0.3	[W/(m*k)]
Thermal conductivity	Water	0.5	[W/(m*k)]
<u>Perfusion</u>			
Density	Tissue	1030	[kg/m^3]
Specific heat	Tissue	3617	[J/(kg*K)]
Perfusion rate	Tissue	0.008	[1/s]
Ambient Temperature	Tissue	37	[°C]

Transducer Efficiency

Duty Cycle During 16 Second Pulse ($\frac{on}{off}$)

Power Per Channel of the IGT Amplifier System

Maximum Amplitude Percent Used Converted to Power Percent

$$TIC = \frac{W}{40D} = \frac{3\frac{W}{channel} * 1000\frac{mW}{W} * 128\ channels * 0.60 * \frac{250ms}{2000ms} * 0.17^2}{40 * 3.7cm} = 5.6$$

Effective Diameter of the Skull Ignoring Curvature

Supplementary Figure 2. TIC calculation based on aperture interacting with the skull
